# PARTICIPANT AND CAREGIVER VIEWS OF SELF-DIRECTION OF HOME AND COMMUNITY SERVICES 15 YEARS AFTER CASH & COUNSELING

**DOI:** 10.1093/geroni/igz038.858

**Published:** 2019-11-08

**Authors:** Kevin J Mahoney, Michelle Putnam

**Affiliations:** 1 Boston College School of Social Work National Resource Center (NRCPDS), Chestnut Hill, Massachusetts, United States; 2 Simmons College School of Social Work, Boston, Massachusetts, United States

## Abstract

Results of the Cash and Counseling controlled experiment are now more than ten years old. This symposium, based on a recent Special Issue of the Journal of Gerontological Social Work on self direction of home and community-based services and supports for people with disabilities, begins with an overview of the status of self-direction in the United States where now over 1.1 million people are managing their own services and supports. After summarizing the six research studies in this Special Issue presenting feedback on the self-direction model from participants, their caregivers, and unpaid representatives followed by participant views on remaining unmet needs, the ideal and undesired characteristics of support brokers, and a research study to develop modules for training care managers and support brokers on person-centered planning and self-direction, papers will be presented focusing on two of these studies highlighting improvements needed in the self-direction approach if it is to become available to all persons with disabilities. The first paper is titled, “Unmet Needs Even When People Have Control of the HCBS Budget”; the second deals with the “Tasks and Characteristics of Supportive Support Brokers”; while the third paper looks at “Present Efforts and Recommendations for Training Support Brokers on the Principles, Values, and Skills to Assist People with Disabilities Who Wish to Direct Their Own Supports”. The session ends with a presentation on the program and policy implications of this research for federal agencies.

